# Short-term results of gait analysis with the Heidelberg foot measurement method and functional outcome after operative treatment of ankle fractures

**DOI:** 10.1186/s13047-021-00505-4

**Published:** 2022-01-08

**Authors:** Jessica C. Böpple, Michael Tanner, Sarah Campos, Christian Fischer, Sebastian Müller, Sebastian I. Wolf, Julian Doll

**Affiliations:** 1 Center for Orthopedics, Trauma Surgery and Spinal Cord Injury, Heidelberg University Hospital, Schlierbacher Landstrasse 200a, 69118 Heidelberg, Germany; 2 ATOS Clinic Heidelberg, Bismarckstr. 9-15, 69115 Heidelberg, Germany

**Keywords:** American orthopedic foot and ankle society hindfoot score, Foot kinematics, Gait analysis, Heidelberg foot measurement method; ankle fractures, Operative treatment

## Abstract

**Background:**

Ankle fractures are common fractures in trauma surgery. Several studies have compared gait patterns between affected patients and control groups. However, no one used the Heidelberg Foot Measurement Method in combination with statistical parametric mapping of the entire gait cycle in this patient cohort. We sought to identify possible mobility deficits in the tibio-talar joint and medial arch in patients after ankle fractures as a sign of stiffness and pain that could result in a pathological gait pattern. We focused on the tibio-talar flexion as it is the main movement in the tibio-talar joint. Moreover, we examined the healing progress over time.

**Methods:**

Fourteen patients with isolated ankle fractures were included prospectively. A gait analysis using the Heidelberg Foot Measurement Method was performed 9 and 26 weeks after surgery to analyse the tibio-talar dorsal flexion, the foot tibia dorsal flexion, the subtalar inversion and the medial arch as well as the cadence, the walking speed and the ground reaction force. The American Orthopedic Foot & Ankle Society ankle hindfoot score was used to obtain clinical data. Results were compared to those from 20 healthy participants. Furthermore, correlations between the American Orthopedic Foot & Ankle Society hindfoot score and the results of the gait analysis were evaluated.

**Results:**

Statistical parametric mapping showed significant differences for the Foot Tibia Dorsal Flexion for patients after 9 weeks (53–75%: *p* = 0.001) and patients after 26 weeks (58–70%: *p* = 0.011) compared to healthy participants, respectively. Furthermore, significant differences regarding the tibio-talar dorsal flexion for patients 9 weeks after surgery (15–40%: *p* < 0.001; 56,5–70%: *p* = 0.007; 82–88%: *p* = 0.033; 97–98,5%: *p* = 0.048) as well as patients after 26 weeks (62,5–65%: *p* = 0.049) compared to healthy participants, respectively. There were no significant differences looking at the medial arch and the subtalar inversion. Moreover, significant differences regarding the ground reaction force were found for patients after 9 weeks (0–17%: *p* < 0.001; 21–37%: *p* < 0.001; 41–54%: *p* < 0.001; 60–64%: *p* = 0.013) as well as patients after 26 weeks (0–1,5%: *p* = 0.046; 5–15%: *p* < 0.001; 27–33%: *p* = 0.001; 45–49%: *p* = 0.005; 57–59%: *p* = 0.049) compared to healthy participants, respectively. In total, the range of motion in the tibio-talar joint and the medial arch was reduced in affected patients compared to healthy participants. Patients showed significant increase of the range of motion between 9 and 26 weeks.

**Conclusions:**

This study shows, that patients affected by ankle fractures show limited mobility in the tibio-talar joint and the medial arch when compared to healthy participants. Even though the limitation of motion remains at least over a period of 26 weeks, a significant increase can be recognized over time. Furthermore, if we look at the absolute values, the patients’ values tend to get closer to those of the control group.

**Trial registration:**

This study is registered at the German Clinical Trials Register (DRKS00023379).

## Background

Fractures of the ankle joint are some of the most common fractures in orthopedic trauma surgery [1–3]. They account for 9% of all fractures and have an incidence of **1**:1000 with a steady increase [4–7]. According to Kannus et al., this increase is so rapid that there could be three times more low-trauma ankle fractures in elderly people in Finland in the year 2030 than in 2000 [8]. Affected patients often suffer from swelling, stiffness, pain and reduced mobility after surgery [9]. An anatomically correct reduction after operative treatment is considered to be essential in preventing long-term consequences such as chronic instability, cartilage damage and early osteoarthritis [10, 11]. Even small joint gaps, axis deviations or instabilities may lead to considerable dysfunction and pain, thereby increasing the risk of post-traumatic arthrosis [12]. As a consequence, a pathological gait pattern can develop [13–15]. For the evaluation of the outcome after surgery in regards to function and pain, different scores such as the American Orthopedic Foot and Ankle Society ankle hindfoot Score (AOFAS) and the Olerud–Molander Ankle Score (OMAS) [16–19] can be used. Although these scores might give a good assessment of the function and patient-reported outcome, they are still quite subjective [17]. As shown in other studies, plain radiographs or computed tomography (CT) are the main instruments to evaluate the consolidation progress of fractures, but they are not able to evaluate the biomechanics and the function of the ankle joint [20–22].

For this purpose, a three-dimensional gait analysis can be used to collect objective information about the gait pattern. Furthermore, it can provide a more reliable predictor of patient-reported functional outcome [7].

By using a detailed, multi-segmented foot model like the Heidelberg Foot Measurement Method (HFMM) or the Oxford Foot Model, kinematic measurements are standardized and more reliable with low inter-rater and stride-to-stride variations, providing reproducible and objectifiable information about gait changes [23, 24]. According to Simon et al. [23] the HFMM can be used to examine both pathological and normal feet. Furthermore, this method accurately reflects the anatomical situation of the ankle joint [23]. Additionally, by using projection angles, rotational angles can be defined independently of any rigid segments, so that the motion in the ankle joint can be observed independently of the forefoot [23]. Several studies to date have analyzed gait patterns of patients with surgically treated ankle fractures without using detailed, multi-segmented foot models such as the HFMM, which is more accurate based on the projection angles. These studies reported differences in gait pattern and function between affected patients with deteriorated ankle kinematics and healthy participants [1, 17, 25–27]. Some of these studies showed a limitation of dorsiflexion as well as plantarflexion between the tibia and the hindfoot in patients with fractured ankles compared to healthy participants [1, 7, 25]. Moreover, Losch et al. [25] and van Hoeve et al. [7] showed a significant slower walking speed in affected patients, while Wang et al. [1] only found a reduced, but not significantly lower walking speed. We wanted to investigate the results of these studies with the HFMM and, if possible, substantiate them. Furthermore, as far as the authors know, there is neither a study on ground reaction force nor a study using statistical parametric mapping (SPM) in patients with ankle fractures.

The aim of the present study was to test our hypothesis that movement in the tibio-talar joint, the subtalar inversion and the medial arch is reduced in affected patients compared to healthy participants. In order to have a better understanding of the impact on gait pattern after ankle fractures and more accurate analysis, we wanted to investigate the abovementioned parameters in more detail using SPM. Additionally, we wanted to determine whether the walking speed, the cadence and the total ground reaction force, a parameter for the plantarflexion moment, are reduced like suggested. Furthermore, we wanted to investigate the healing progress over time by looking at the changes of the range of motion, speed and ground reaction force as well as using the AOFAS to measure patient-reported outcomes over time.

## Patients and methods

### Ethics

This prospective monocentric controlled study was approved by the local ethics committee (S-402/2009), registered at the German Clinical Trials Register (DRKS00023379) and conducted in accordance with the declaration of Helsinki in its current form. All individuals agreed with the study protocol and gave their written informed consent.

### Patients

The study was performed over a 3-year period (from 09/2009 to 09/2011) at our Centre for Orthopedics, Trauma Surgery and Spinal Cord Injury. A total of 18 patients with appropriate matching criteria were recruited prospectively.

Only patients over the age of 18 with an any type of an isolated unilateral ankle fracture and scheduled operative treatment, a healthy contralateral leg without any known illnesses and uninhibited ability to walk were included.

Exclusion criteria were injuries, previous surgeries or pathological alterations of the lower extremities, not including the ankle fracture itself and consequences thereof (e.g. surgery, postoperative infections or deficits). Patients with neurological diseases, deficits and conditions that impair gait and sense of balance were also excluded.

The fractures were diagnosed by an anteroposterior and lateral radiograph. Each patient was operated by an experienced, board-certified trauma surgeon within 8 days after injury. Fixation was performed with plate and screw osteosyntheses according to AO-principles [28].

The ankle was then immobilized in a cast for 6 weeks. During this time, partial weight-bearing with 20 kg was allowed, supported by physiotherapy and manual lymphatic drainage. Thereafter, weight-bearing was increased to full weight-bearing over a period of 3 weeks.

Study patients passed a follow-up of 26 weeks which included clinical and radiological examinations.

The participants of the control group, which had no abnormalities in the lower limbs or feet, were chosen in order for the age to be similar between groups. Both feet of the control group (*n* = 40) were used to compare the outcomes between these two groups.

### Study protocol

**Fig. 1:**
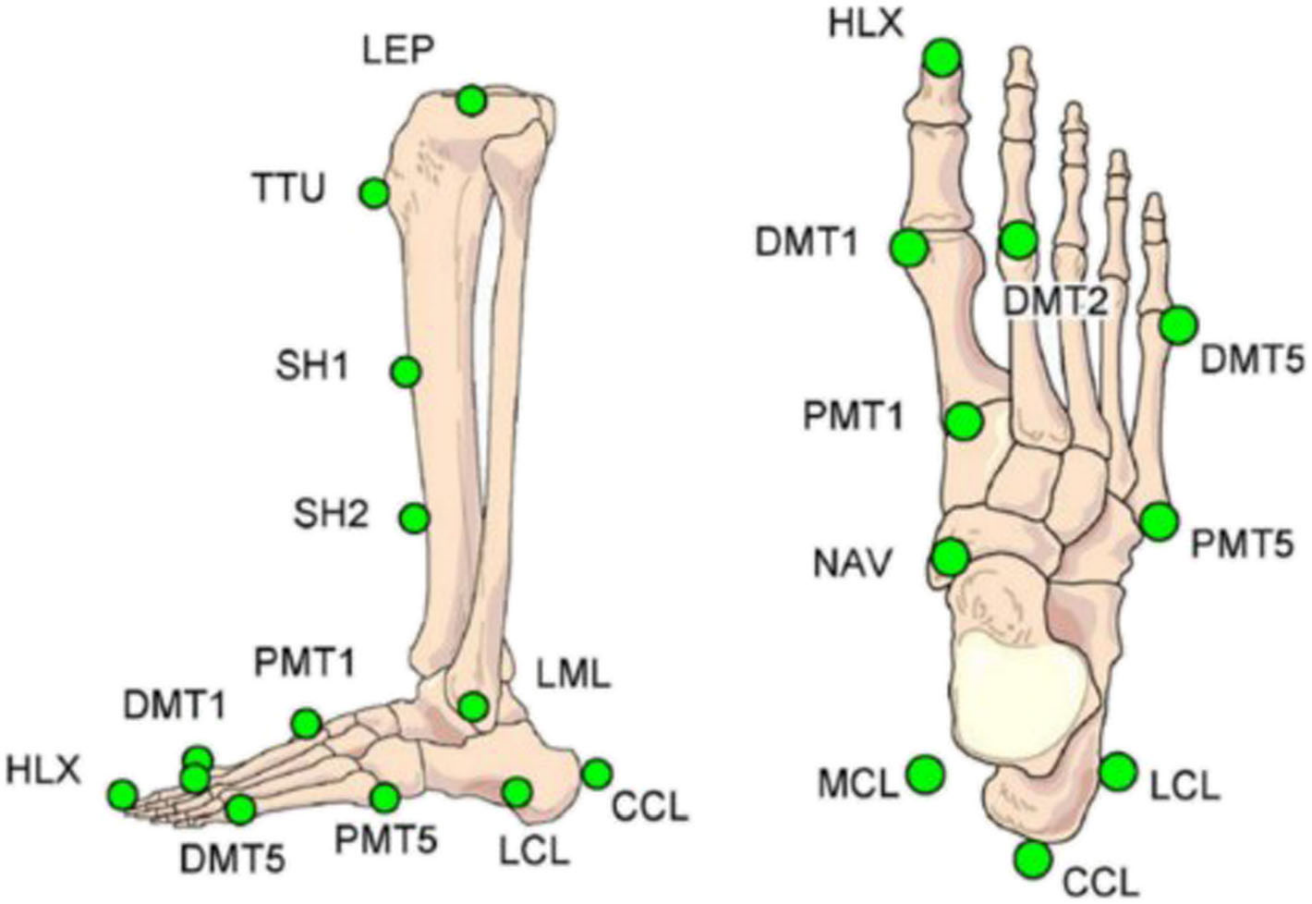
Marker placement. Abbreviations mentioned in Table 3 [23]

Instrumented 3D gait analysis including the HFMM was performed after full weight-bearing had been achieved at 9 and 26 weeks postoperatively [29]. A total of 17 (5 on the knee, 12 on the foot) retro-reflective markers are placed on defined bony landmarks of both lower limbs in this model according to Simon et al. [23] (Fig. 1, Table 1). Additional markers were placed on the pelvis according to standard procedures for instrumented 3D gait analysis [30] in order to evaluate the joint kinematics of hip, knee and ankle, respectively. The combination of the two marker-sets allows the identification of potential compensations mechanisms after an operated ankle fracture. Data collection of marker coordinates was performed at 120 Hz with a 12 camera VICON motion capture system (Oxford Metrics Inc., Oxford, UK) using the standard Y-X-Z cardan sequence (sagittal, frontal and transverse respectively). Further, ground reaction forces were detected by means of KISTLER force plates (KISTLER Instrumente AG, Winterthur, Switzerland) normalized to the gait cycle (101 data points) and the body weight (kg) of the individual patient/ healthy subject.

**Table 1:** Description of the marker placement of the Heidelberg Foot Measurement Method [23]

Marker labelling	Description
LEP and MEP	Lateral and medial of the knee at the estimated knee flexion axis
TTU	most prominent part of tibial tuberosity
SH1 and SH2	2 points on the medial surface of the tibia avoiding contact to foot extensor muscles and dividing the tibia into approximately three equal parts
LML and MML	Lateral and medial malleolus, placed such that the line through the markers determines the largest distance
MCL and LCL	Medial and lateral point on the calcaneus defined by the heel alignment device as described in the text
CCL	Placed dorsal on the calcaneus at the landmark at the insertion of the Achilles tendon
NAV	Placed on the navicular such that in the frontal view the marker axis is seen at 45° to the floor. In the case that the foot extensor tendon interferes, this marker has to be placed more medially
PMT1	Joint gap between first cuneiform and MT I placed such that in the frontal view the marker axis is seen at 45° to the floor
DMT1	Head of MT I at 45° angle between marker axis and floor
HLX	Midpoint of the distal phalanx of hallux
DMT2	Head of MT II DMT5 Head of MT V at 45° angle between marker axis and floor
PMT5	Tuberositas ossis MT V

Using the reflective markers on the foot, the following angles and movements were determined: tibio-talar-flexion, medial arch inclination, medial and lateral arch angle, subtalar inversion, forefoot/ankle supination, forefoot/midfoot supination, forefoot/hindfoot abduction, forefoot/ankle abduction, inter metatarsal I-V angle, hallux adduction and hallux flexion (for more detailed information see Table 2). Missing values were supplemented with pre-set filling options in Nexus 2.12 (Vicon, Oxford Metrics Inc., Oxford, UK). The VCM (Vicon Clinical Manager; Oxford Metrics Inc., Oxford, UK) spline low-pass filter was used for kinematic data, whereas the Butterworth filter was used for kinetic data. Before all patients were asked to walk a 7-m path barefoot with a self-selected walking speed, a static measurement in a standing posture was performed as reference. The starting point was determined and adjusted in case the patient did not hit the force plates embedded in the floor correctly. Experienced physiotherapists and biomedical engineers performed all assessments according to standardized protocols with quality control.

**Table 2:** Angles of the Heidelberg Foot Measurement Method [23]

Angle	Description
Tibio-talar flexion	Flexion between tibia and talus (represented by the calcaneal and navicular motion) as rotation around the malleolar line, approximately sagittal plane
Medial arch angle	Absolute angle in 3D between line from medial calcaneus marker to navicular and MT I, approximately sagittal plane
Subtalar inversion	Rotation of calcaneus around subtalar axis, approximately frontal plane

Data from ten strides per patient were collected, averaged and evaluated on the basis of the HFMM. Further, time distance parameters such as speed and cadence were determined.

Furthermore, the “American Orthopedic Foot and Ankle Score (AOFAS)”, a viable indicator for clinical changes in foot and ankle studies including questions regarding pain, function and alignment [31, 32], was collected at these intervals.

### Statistical analysis

We wanted to examine the exact changes in the range of motion of tibio-talar dorsal flexion, foot tibia dorsal flexion, subtalar inversion and medial arch between 9 weeks and 26 weeks after surgery as well as the differences compared to healthy participants. In addition, we wanted to investigate the same changes and differences in cadence, walking speed and total ground reaction force (GRF).

Statistical calculations were performed using Stata statistical software (version 16.1, StataCorp, Texas, United States). Joint angles were calculated with Matlab R2018b (v9.5.0.944444) and MoMo (MotionModeller) as described by Simon et al. [23]. Means and standard deviations were calculated. One dimensional statistical parametric mapping (SPM) was performed with ANOVA-1D using Matlab R2018b (v9.5.0.944444) to compare the biomechanical outcomes throughout the whole gait cycle.

Owing to the small sample size in each group, we assumed that data might not to be normally distributed, which is why we applied the Wilcoxon signed rank test to compare both groups. A Fisher’s exact test was used to compare categorical variables between groups. The patient’s characteristics and categorial variables were analyzed using descriptive statistics and the two-sample t-test. To compare patients after 9 and 26 weeks we used a paired t-test. The repeated measures correlation reported from Bakdash et al. [33] was performed to identify associations between AOFAS and gait analysis parameters. Regarding the correlation analysis a Bonferroni correction was performed to adjust for multiple testing. In all statistical tests, an effect with a *p*-value below 0.05 was considered statistically significant.

### Post-hoc power analysis

A post-hoc power analysis for differences between the control and intervention group at 9 weeks was performed. We reached a power of 99.9% for tibio-talar dorsal flexion, the foot tibia dorsal flexion, the subtalar inversion and the medial arch respectively.

## Results

### Patient characteristics

**Fig. 2:**
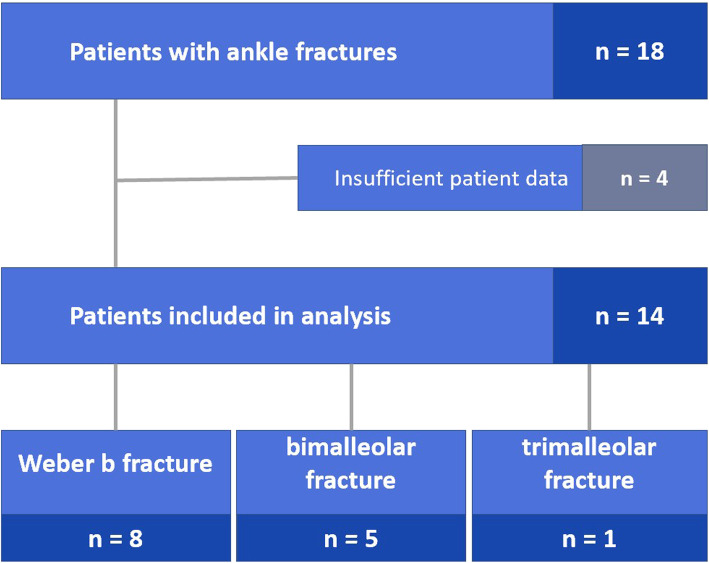
Study flow

We enrolled a total of 18 patients at the Heidelberg University Hospital Centre for Orthopedics, Trauma Surgery and Spinal Cord Injury over a period of 3 years. Four study patients had to be excluded as they were lost to follow-up. Of the 14 remaining patients, 8 patients had an isolated Weber B fracture, 5 patients had a bimalleolar fracture and 1 patient had a trimalleolar fracture (Fig. 2). Every single patient showed signs of complete consolidation on X-rays 26 weeks after surgery. For statistical comparison, 20 healthy participants were included into the control group (*n* = 40 ft). There were no significant differences between the demographic data of both groups as they were matched (Table 3).

**Table 3:** Demographic data of the control group in comparison to the intervention group

	CG	IG (9 weeks)	p-value
n	20	14	
age, in years	47.2 ± 10.4	50.9 ± 16.2	0.23
height, in cm	174.0 ± 9.1	171.0 ± 7.6	0.27
weight, in kg	73.0 ± 14.0	78.5 ± 13.6	0.26
gender			0.27
*male*	9	3	
*female*	11	11	

abbreviations: CG = control group; IG = intervention group; SD = standard deviation; *p* ≤ 0.05 considered as statistically significant, values are presented as mean ± SD as appropriate

### Kinematics

#### Mobility of the Tibio-Talar ankle joint

**Fig. 3:**
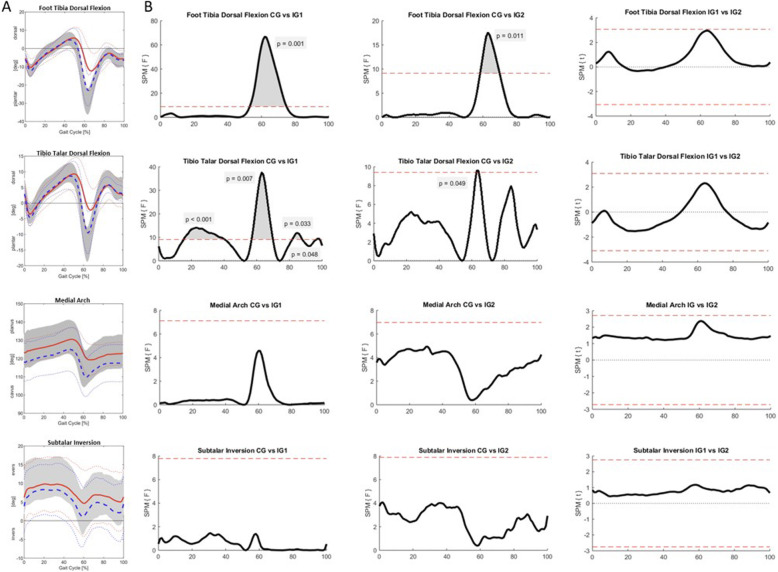
Foot kinematics and statistical parametric mapping. **A** foot kinematics of the tibio-talar dorsal flexion, foot tibial dorsal flexion,medial arch and subtalar inversion over the whole gait cycle shown for the intervention group after 9 weeks (red solid line) and 26 weeks (blue dashed line) as well as for the control group (grey bar); thin dotted color-coded lines are the 95% quantiles; **B** every column represents the comparison of two clinical groups (first column: control group versus intervention group after 9 weeks; second column: control group versus intervention group after 26 weeks; third column: intervention group after 9 weeks versus intervention group after 26 weeks). Every line represents one angle with the different comparisons

Figure 3 shows the movement in the different joint for the whole gait cycle as well as the results of SPM. Table 4 presents the values of the range of motion (ROM) in all three joints from the patients with ankle fractures and from the control group.

**Table 4:** Values of the tibio-talar dorsal flexion, foot tibial dorsal flexion and medial arch. ROM over the whole gait cycle shown for the intervention group after 9 weeks (IG1) and 26 weeks (IG 2) as well as for the control group (CG); *p* ≤ 0.05 considered as statistically significant; values are presented as median (IQR) as appropriate

	CG	IG 1	IG 2	p-value CG vs IG1	p-value CG vs IG2	p-value G1 vs IG2
n	40	14	14			
Tibio-Talar Dorsal Flexion, in degree	24.8 (19.6–29)	15.9 (11.439–18.4)	18.3 (16.9–19.8)	< 0.001	< 0.001	0.081
Foot Tibia Dorsal Flexion, in degree	36 (30.2–38.8)	21.3 [17–23]	26.8 (24.4–31)	< 0.001	< 0.001	0.004
Subtalar Inversion, in degree	11.1 (9.4–13.5)	6.7 (5.3–7.7)	8.9 (7.5–11.1)	< 0.001	0.018	0.009
Medial Arch,in degree	18 (16.5–20.1)	12.5 (9.85–15.5)	16.3 (14.6–18.6)	< 0.001	0.009	0.027

#### Tibio-Talar dorsal flexion (dorsal extension/ plantar flexion)

The tibio-talar dorsal flexion describes the exact range of motion in the tibio-talar joint.

Statistical parametric mapping showed significant differences between the healthy participants and the patients after 9 weeks over approximately 15–40% (*p* < 0.001), 56–70% (*p* = 0.007), 82–88% (*p* = 0.033) and 97–98% (*p* = 0.048). The critical threshold here (red dashed line) was 9. Furthermore, significant differences between the healthy participants and the patients after 26 weeks over approximately 62–65% (*p* = 0.049) could be found. The critical threshold (red dashed line) was 9. Between the patients after 9 and after 26 weeks there was no significant difference with a critical threshold of 3.

In comparison to the control group a significant lower ROM 9 weeks (15.9° (IQR: 11.3–18.4) vs 24.8° (IQR: 19.6–29), *p* < 0.001) and 26 weeks (18.3° (IQR: 16.9–19.8) vs 24.8° (IQR: 19.6–29), *p* < 0.001) after surgery, could be observed. Additionally, there was no significant increase of the ROM between 9 and 26 weeks after surgery (15.9° (IQR: 11.3–18.4) vs 18.3° (IQR: 16.9–19.8), *p* = 0.081).

#### Foot tibia dorsal flexion (dorsal extension/ plantar flexion)

The foot tibia dorsal flexion describes the entire range of motion of the foot in relation to the tibia around the axis build by the malleoli.

Statistical parametric mapping showed significant differences between the healthy participants and the patients after 9 weeks over approximately 53–75%(*p* = 0.001). The critical threshold here (red dashed line) was 9. Furthermore, significant differences between the healthy participants and the patients after 26 weeks over approximately 58–70% (*p* = 0.011) could be found. The critical threshold (red dashed line) was 9. Between the patients after 9 and after 26 weeks there was no significant difference with a critical threshold of 3.

Compared to the control group significant differences 9 weeks (21.3° (IQR: 17–23) vs 36° (IQR: 30.2–38.8), *p* < 0.001) and 26 weeks (26.8*°* (IQR: 24.4–31) vs 36° (IQR: 30.2–38.8), *p* < 0.001) after surgery could be detected with lower values in patients with fractures. There was also a significant difference between the mean ROM after 9 and after 26 weeks (21.3° (IQR: 17–23) vs 26.8*°* (IQR: 24.4–31), *p* = 0.004).

#### Subtalar inversion

The subtalar inversion is a parameter indicating the rotation of calcaneus around subtalar axis.

Statistical parametric mapping showed no significant differences between the healthy participants and the patients after 9 weeks as well as the patients after 26 weeks. The critical thresholds (red dashed line) were 8 after 9 weeks and 8 after 26 weeks. Between the patients after 9 and after 26 weeks there was no significant difference with a critical threshold of 3.

Between 9 weeks and 26 weeks after surgery a significant increase of the mobility (6.7° (IQR: 5.3–7.7) vs 8.9° (IQR: 7.5–11.1), *p* = 0.009), indicated by the subtalar inversion, could be observed. In comparison to the control group there was a significant difference to the patients after 9 weeks (6.7° (IQR: 5.3–7.7) vs 11.1° (IQR: 9.4–13.5), *p* < 0.001) and after 26 weeks (8.9° (IQR: 7.5–11.1) vs 11.1° (IQR: 9.4–13.5), *p* = 0.018).

#### Medial arch

The medial arch is a parameter indicating the flexibility of the arch of the foot. Statistical parametric mapping showed no significant differences between the healthy participants and the patients after 9 weeks as well as the patients after 26 weeks. The critical thresholds (red dashed line) were 7 after 9 weeks and 7 after 26 weeks. Between the patients after 9 and after 26 weeks there was no significant difference with a critical threshold of 3.

Between 9 weeks and 26 weeks after surgery a significant improvement of the mobility (12.5° (IQR: 9.85–15.5) vs 16.3° (IQR: 14.6–18.6), *p* = 0.027), indicated by the medial arch, could be observed. In comparison to the control group there was a significant difference to the patients after 9 weeks (12.5° (IQR: 9.85–15.5) vs 18° (IQR: 16.5–20.1), *p* < 0.001) and after 26 weeks (16.3° (IQR: 14.6–18.6) vs 18° (IQR: 16.5–20.1), *p* = 0.009).

### Time-distance parameters

In Table 5 the values for walking speed and cadence of the ankle fractures patients and the control group are presented.

**Table 5:** Values of cadence and speed. Values over the whole gait cycle shown for the intervention group after 9 weeks (IG1) and 26 weeks (IG 2) as well as for the control group (CG); p ≤ 0.05 considered as statistically significant; values are presented as median (IQR) as appropriate

	CG	IG 1	IG 2	*p*-value CG vs IG1	*p*-value CG vs IG2	*p*-value G1 vs IG2
n	20	14	14			
Cadence, in steps per minute	115 (109–118)	103 (95.5–111)	110 (108–118)	0.001	0.26	0.039
Speed, in m/s	1.37 (1.18–1.49)	0.92 (0.65–1.08)	1.15 (1.05–1.2)	< 0.001	0.004	0.002

#### Cadence

Cadence is defined as ‘steps per minute made within the walkway’. Considering median cadence in the control group, cadence after surgery showed a significant lower value with *p* = 0.001 (103 steps per minute (IQR: 95.5–111) vs 115 steps per minute (IQR: 109–118)) after 9 weeks but not after 26 weeks (110 steps per minute (IQR: 108–118) vs 115 steps per minute (IQR: 109–118), *p* = 0.26). Additionally, there was a significant difference in steps per minute between 9 and 26 weeks after surgery (103 steps per minute (IQR: 95.5–111) vs 110 steps per minute (IQR: 108–118), *p* = 0.039).

#### Walking speed

Compared to the control group the self-selected speed was significantly lower in the operated group after 9 weeks (0.92 m/s (IQR: 0.65–1.08) vs 1.37 m/s (IQR: 1.18–1.49), *p* < 0.001) and after 26 weeks (1.15 m/s (IQR: 1.05–1.2) vs 1.37 m/s (IQR: 1.18–1.49), *p* = 0.004). There was a significant difference in speed between 9 and 26 weeks after surgery (0.92 m/s (IQR: 0.65–1.08) vs 1.15 m/s (IQR: 1.05–1.2), *p* = 0.002).

### Ground reaction force (GRF)

**Fig. 4:**
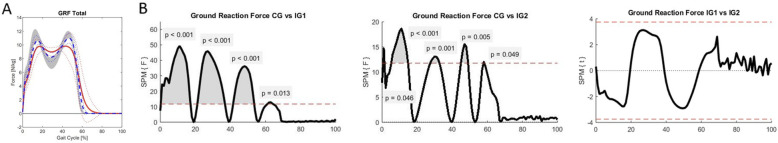
Ground reaction force and statistical parametric mapping. **A** GRF over the whole gait cycle shown for the intervention group after 9 weeks (red solid line) and 26 weeks (blue dashed line) as well as for the control group (grey bar); thin dotted color-coded lines are the 95% quantiles; **B** every column represents the comparison of two clinical groups (first column: control group versus intervention group after 9 weeks; second column: control group versus intervention group after 26 weeks; third column: intervention group after 9 weeks versus intervention group after 26 weeks)

Statistical parametric mapping showed significant differences between the healthy participants and the patients after 9 weeks over approximately 0–17% (*p* < 0.001), 21–37% (*p* < 0.001), 41–54% (p < 0.001) and 60–64% (*p* = 0.013). The critical threshold here (red dashed line) was 11.72. Furthermore, significant differences between the healthy participants and the patients after 26 weeks over approximately 0–1,5% (*p* = 0.046), 5–15% (p < 0.001), 27–33% (*p* = 0.001), 45–49% (*p* = 0.005) and 57–59%: (*p* = 0.049) could be found. The critical threshold (red dashed line) was 11.82 (Fig. 4). Between the patients after 9 and after 26 weeks there was no significant difference with a critical threshold of 3.75.

There was a significant increase of the GRF between 9 weeks and 26 weeks after surgery (*p* = 0.008). Compared to the control group the maximum GRF was significantly lower after 9 weeks (*p* < 0.001) as well as after 26 weeks (*p* < 0.001) (Table 6).

**Table 6:** Values of the GRF” values over the whole gait cycle shown for the intervention group after 9 weeks (IG1) and 26 weeks (IG 2) as well as for the control group (CG); *p* ≤ 0.05 considered as statistically significant; values are presented as median (IQR) as appropriate.

	CG	IG 1	IG 2	*p*-value CG vs IG1	*p*-value CG vs IG2	*p*-value IG1 vs IG2
n	40	14	14			
GRF, in N/kg	11.7 (11.3–12.5)	10.2 (10–10.6)	11 (10.7–11.4)	< 0.001	< 0.001	0.008

### AOFAS score and correlations

A significant improvement of the AOFAS score between both groups from follow-up appointments at 9 to 26 weeks could be observed (*p* = 0.0058). The AOFAS Score was 68 (IQR 62–85) 9 weeks after surgery and 87 (74–95) 26 weeks after surgery.

**Fig. 5:**
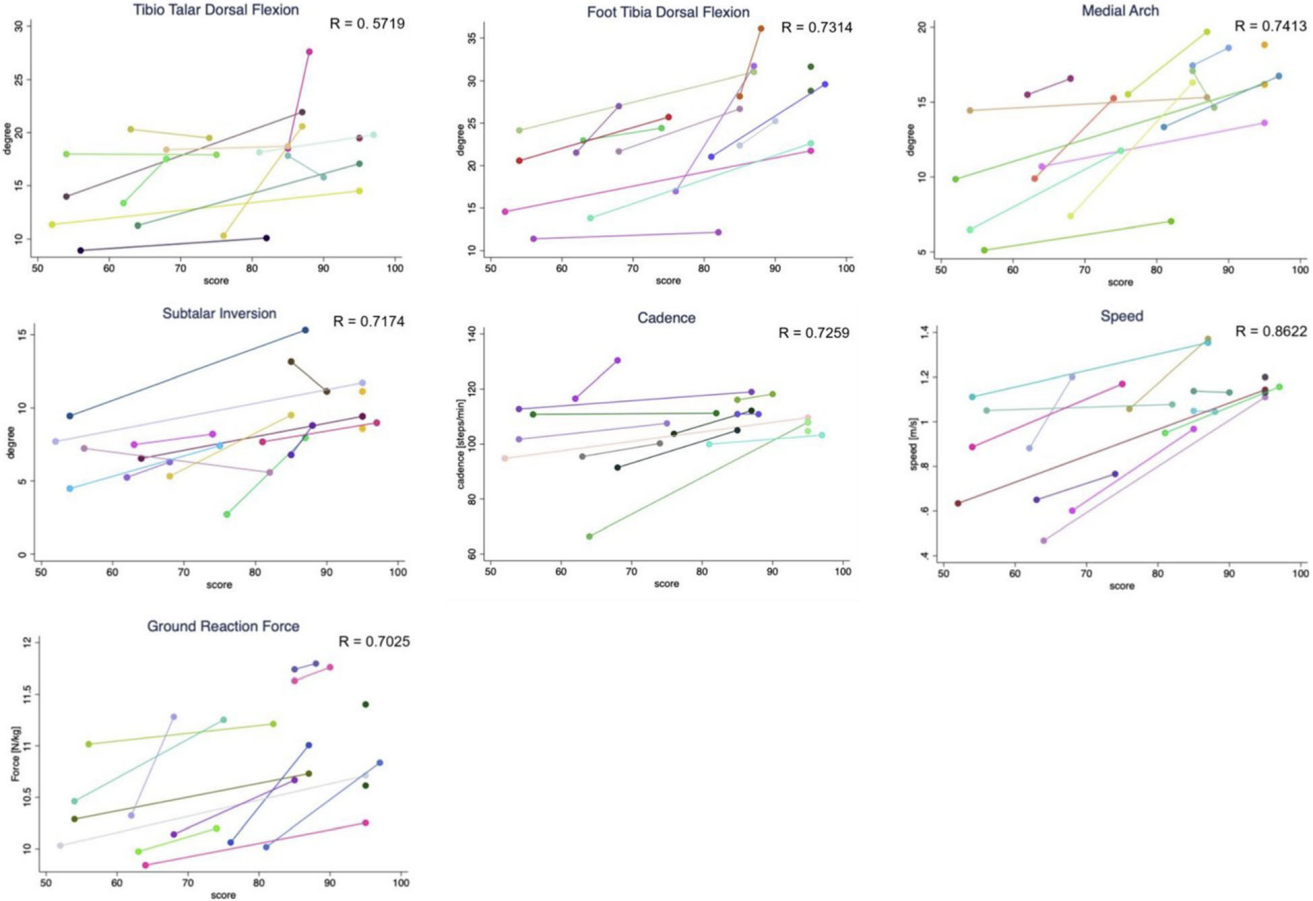
Repeated Measures Correlations between gait analysis parameters and AOFAS

For correlation analyses, data for both time points (9 and 26 weeks after surgery) of 13 patients with ankle fractures were included. One could not be included due to missing data in the AOFAS score. A significant and strong correlation was found between the AOFAS Score and the ROM of foot tibia dorsal flexion (R = 0.7314, *p* = 0.021), the subtalar inversion (R = 0.7174, *p* = 0.0273), the medial arch (R = 0.7413, *p* = 0.0168), GRF (R = 0.7025, *p* = 0.0357), cadence (R = 0.7259, *p* = 0.0231) as well as speed (R = 0.8622, *p* = 0.0007). There was no significant correlation between the AOFAS Score and tibio-talar dorsal flexion (R = 0.5719, *p* = 0.2282) (Fig. 5).

## Discussion

Management of ankle fractures and their anatomically correct reduction after operative treatment is considered to be essential in preventing long-term consequences [11] and is often underestimated [34]. Accordingly, an objective diagnostic tool such as the three-dimensional gait analysis is rewarding to collect more information about biomechanics and the function in the ankle joint [1].

Although some studies already focused on gait patterns after operatively treated ankle fractures, none of them evaluated the gait cycle using statistical parametric mapping. Moreover, parameters like walking speed and ground reaction force are still controversially discussed. The aim of this study was to objectify possible reduced mobility of the tibio-talar joint and the medial arch 9 weeks and 26 weeks after surgery using the HFMM for gait analysis and applying statistical parametric mapping to evaluate not only the range of motion in general but also the changes of the angle throughout a gait cycle.

The most important findings of this study were the differences detected using statistical parametric mapping. Significant differences could be found looking at the tibio-talar dorsal flexion, the foot tibia dorsal flexion as well as the GRF for patients after 9 and 26 weeks compared to healthy participants, respectively. In addition, a significantly smaller range of motion in dorsiflexion and plantarflexion in the foot tibia dorsal flexion as well as in the tibio-talar dorsal flexion after surgery could be seen. Furthermore, we found a smaller range of motion of the medial arch as a parameter of the sagittal plane. Additionally, walking speed significantly differed between all groups. Moreover, we found a significant improvement of the AOFAS from 9 weeks to 26 weeks after surgery.

Previous studies already showed a restriction of clinical dorsiflexion and plantarflexion between the tibia and the hindfoot in the stance and swing phase in affected patients compared to healthy participants [1, 7, 25]. This is in line with our results of restricted movement in the tibio-talar joint. Furthermore, we were able to show that these results are the same regardless of the foot model applied. While Wang et al. [1] and van Hoeve et al. [7] used the Oxford foot model or a modified version of it with just 3 segments for their examinations we used the HFMM. When comparing the results, one has to consider that the HFMM takes the independent movements of the tibio-talar and subtalar joint into account, while the Oxford foot model imagines these two just as a ball-and-socket joint. This may lead to a discrepancy regarding the values of the motion in both joints. The marker placement of the HFMM enables a detailed measurement of the mobility in the tibio-talar joint unaffected of any movements in adjoining joints, by looking at the values of tibio-talar dorsal flexion [23].

Furthermore, this study showed a significant slower walking speed when the patients were asked to walk with their self-selected speed. This coincides with the results of Losch et al. [25] and van Hoeve et al. [7], who also found a significant difference between the walking speed of the affected and non-affected participants. In contrast, Wang et al. [1] only found a slightly, but not significantly lower walking speed. Compared to our results, the difference can be explained by the different time points between examinations and the surgery with 9 weeks or 26 weeks compared to 1 year after surgery. Regarding the walking speed, one should also look at the study by Fukuchi et al. [35] and Stoquart et al. [36]. Here a moderate correlation between plantar flexion and gait speed was shown. Thus, an influence of gait speed on our sagittal ankle angles would be conceivable. However, the effects on knee flexion were highest in these studies [35, 36]. All in all, the influence of gait speed should not be underestimated.

Additionally, Wang et al. [1] surmised that affected patients tend to lift the foot rather than pushing it off. Regarding the statistical parametric mapping of the GRF we could prove this hypothesis. The values 9 weeks after surgery are the lowest, perhaps because the patients want to relieve their injured foot as a compensation mechanism. On the other hand, the restriction of GRF might result from some anatomical issues. According to Nagai et al. [37] and Hayashi et al. [38], immobilization of joints after injuries can lead to muscle atrophy as well as reduction of muscle extensibility. Our patients were both immobilized in a cast for 6 weeks and restricted regarding weight-bearing. Due to the resulting muscle atrophy of the calf muscles a reduced mobility in the joints of their foot was developed. As they are important for the propulsive force [39], this can lead to a reduced GRF. By full weight-bearing, the muscles expand again and reassume prior size. This could be the reason for the higher values for the GRF at the 26 weeks appointment compared to 9 weeks. Furthermore, a higher GRF coincides with an increase in walking speed [40, 41], which also significantly improved in our patients over time.

Looking at the AOFAS score, we found a significant improvement between the two time points. So far, no minimal clinically important difference (MCID) has been specified for ankle fractures [42]. However, considering the study of Norman et al. [43] it has been assumed as half the standard deviation (SD). According to studies of syndesmosis injuries and ankle fractures, the SD can be assumed to be 12 points [44, 45]. Due to this, the MCID for the AOFAS score would be 6 points. Given the increase of 19 points, the change in AOFAS score between 9 and 26 weeks after surgery can be considered clinically relevant.

There were certain limitations in our study. For instance, owing to the low sample size, a comparison of the different ankle fractures such as Weber A, B and C was statistically not possible. Additionally, it was not feasible to precisely match each patient, leading to a more inaccurate group matching. Furthermore, marker placement as well as skin motion can result in systematically errors especially in the medial arch, as there is the greatest standard deviation and the lowest test-retest reliability.

## Conclusions

In conclusion, patients affected by ankle fractures showed limited movement in the tibio-talar joint and the medial arch compared to healthy controls. Although this limitation persists both, at 9 and 26 weeks, compared to healthy controls, a significant improvement can be seen over time. A tendency towards the control group can be seen in the absolute values.

Our results could be used to develop future randomized controlled trials or prospective cohort studies based on similar conditions.

## Data Availability

The datasets used and analyzed during the current study are available from the corresponding author on reasonable request.

## Abbreviations

AOFAS: American Orthopedic Foot and Ankle Society hindfoot score
HFMM: Heidelberg Foot Measurement Method
OMAS: Olerud–Molander Ankle Score
